# Catalytic Effect of Amyloid-β on Native Tau Aggregation at Physiologically Relevant Concentrations

**DOI:** 10.3390/ijms262412128

**Published:** 2025-12-17

**Authors:** Rakhi Chowdhury, Apu Chandra Das, Ruan van Deventer, Luda S. Shlyakhtenko, Yuri L. Lyubchenko

**Affiliations:** 1 Department of Pharmaceutical Sciences, University of Nebraska Medical Center, 4040 Emile Street, Omaha, NE 68198-6120, USA; rachowdhury@unmc.edu (R.C.); rvandeventer@unmc.edu (R.v.D.); lshlyakhtenko@unmc.edu (L.S.S.); 2 Department of Biostatistics, University of Nebraska Medical Center, 4040 Emile Street, Omaha, NE 68198-6120, USA; apdas@unmc.edu

**Keywords:** tau, amyloid beta, Alzheimer’s disease, aggregation, AFM imaging

## Abstract

Alzheimer’s disease (AD) is characterized by the accumulation and aggregation of tau and amyloid-β (Aβ). The pathophysiology and progression of AD are facilitated by the neurotoxic effects of these aggregated proteins, resulting in neurodegeneration and memory loss. In this context, the interaction between tau and Aβ42 is considered, but the mechanism underlying their pathogenic interplay remains unclear. Here, we addressed this question by studying the aggregation of full-length, unmodified tau and Aβ42 at physiologically low concentrations using atomic force microscopy (AFM). AFM imaging and data analyses demonstrate an increase in tau aggregation in the presence of Aβ42, characterized by increased sizes and number of aggregates. Importantly, tau aggregation occurs without the need for phosphorylation or any other post-translational changes. The analysis of the data demonstrates that tau and Aβ42 form co-aggregates, with no visible accumulation of Aβ42 aggregates alone. Given that the catalysis of tau aggregation by Aβ42 is observed at physiological low nanomolar concentrations of Aβ42, the finding suggests that such aggregation catalysis of tau by Aβ42 can be a molecular mechanism underlying the pathological tau aggregation process associated with the onset and development of Alzheimer’s disease.

## 1. Introduction

Alzheimer’s disease (AD) is the most common form of dementia, affecting nearly two-thirds of individuals over 65 years of age [1]. Its two major pathological hallmarks are the accumulation of amyloid-beta (Aβ) aggregates [2] and the formation of tau-containing neurofibrillary tangles, particularly in the entorhinal cortex, limbic regions, and associated cortices [3,4,5]. Notably, these changes begin up to two decades before the clinical onset of AD [6], underscoring the importance of understanding the molecular mechanisms that drive tau & Aβ pathology. In their monomeric forms, both tau & Aβ serve essential neuronal functions: Aβ contributes to synaptic activity and plasticity, while tau stabilizes microtubules and maintains cytoskeletal integrity [3,4,5,6]. However, conformational transitions trigger the misfolding of Aβ and tau, which lead to toxic aggregation [5,7]. While tau-containing neurofibrillary tangles have historically defined AD pathology, tau oligomers are increasingly recognized as critical drivers for tau propagation and synaptic disruption. Similarly, oligomeric Aβ species are increasingly linked to neuronal toxicity, suggesting that toxic oligomers for both proteins, rather than their mature aggregates, are central to disease progression [8,9,10,11]. Increasing evidence points to a synergistic relationship between Aβ and tau in AD [11,12,13]. For instance, Aβ deposition correlates with the spread of tau aggregates [11,14], possibly by promoting tau oligomerization, a process linked to synaptic dysfunction, calcium dyshomeostasis, and microtubule destabilization, although the molecular mechanisms remain uncertain [15,16]. In addition, Aβ activates kinases such as CDK-5 and GSK-3β, which are associated with tau hyperphosphorylation and may thereby contribute to tau-mediated neurotoxicity [11]. Collectively, these findings suggest that Aβ and tau aggregation may be mechanistically linked in AD development [15,17,18]; however, the precise molecular basis of their interaction remains unclear.

In this work, we examined the interplay between native full-length tau and Aβ42 at physiologically relevant nanomolar concentrations. Using atomic force microscopy (AFM), we directly visualized Aβ42 and tau aggregation, enabling characterization of the aggregation process. Our findings indicate that Aβ42 at nanomolar levels can accelerate the aggregation of unmodified tau and that the two proteins can co-aggregate.

## 2. Results

### 2.1. Experimental Setup

To mimic the aggregation process of Aβ42 at physiologically relevant environment concentrations, specifically in the low nanomolar range [19], we employed the on-surface aggregation approach [20,21], in which AFM is used to directly visualize aggregates assembled as a function of time on a mica substrate. We placed cleaved mica pieces in protein solutions and incubated them for 2 min, 3 h, and 5 h, after which the mica was rinsed, dried, and imaged with AFM to visualize aggregate formation. Figure S1 provides a schematic of this approach.

### 2.2. AFM Imaging of Tau Aggregation

**Figure 1:**
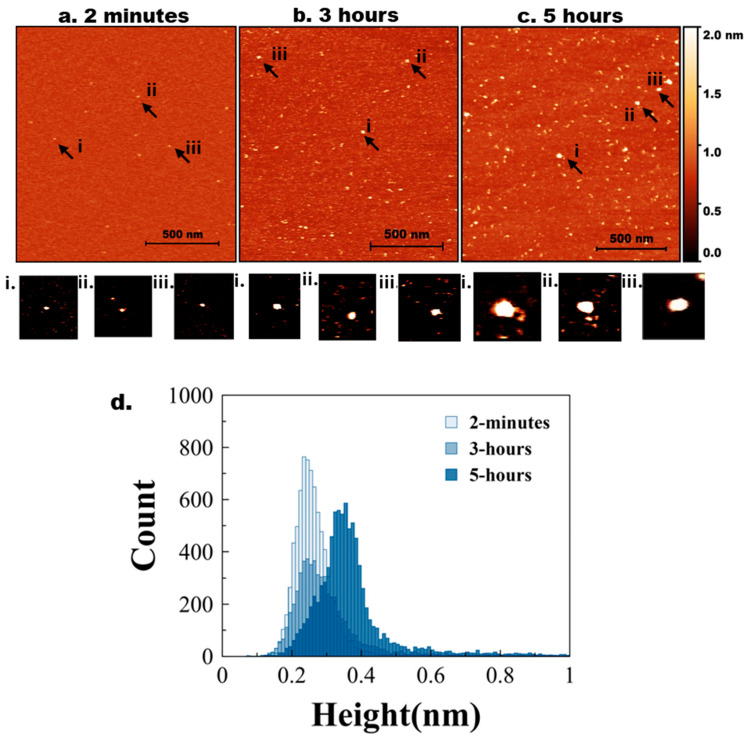
AFM imaging of tau aggregation. Representative AFM topographic images of tau aggregates on mica surfaces at different incubation times. (**a**) 2 min, (**b**) 3 h, (**c**) 5 h. Arrows indicate examples of aggregates. (**a**,**i**–**iii**) represents zoomed-in particles from 2 min. (**b**,**i**–**iii**) represents zoomed-in particles from 3 h. (**c**,**i**–**iii**) represents zoomed-in particles from 5 h. (**d**) Tau height distribution for 2 min, 3 h, and 5 h with baseline shift.

AFM images of mica specimens incubated in a solution of tau protein at 10 nM are shown in Figure 1. Initially (2 min, Figure 1a), only a few particles of round shape were seen. A few zoomed-in images, indicated with arrows labelled i, ii, and iii, are shown below in Figure 1a. As incubation time increased to 3 h (Figure 1b), the number of tau aggregates on the surface increased. Similarly, zoomed images of a few particles (i, ii, and iii) are shown below Figure 1b. These particles are visually larger than those in Figure 1a.

The aggregation is more pronounced after 5 h of incubation (Figure 1c), as illustrated by a few zoomed-in images in Figure 1c(i–iii). Together, these data indicate that over the 5-h incubation period, tau progressively self-assembles into larger aggregates, demonstrating that even unmodified tau can undergo aggregation at low nanomolar concentrations.

The particle size distribution was quantified by measuring the heights of particles appearing on the mica substrates over time. Ten line traces were drawn across the images (Figure S2a), and the variability in particle heights along each line is shown in Figure S2b, with each line represented by a different color. These measurements from different images were used to generate the histograms in Figure 1d, where the light blue and dark blue bars correspond to the 2-min, 3 h, and 5-h incubation times, respectively. Figure S8a shows a similar histogram with three different colors for clear representation. The shift toward larger particle heights at 5 h reflects the progressive aggregation of tau over time. Three independent experiments were conducted. Figure S3 demonstrates the overlap plot of the height traces for 2 min and 5 h, providing a clearer visual representation of data reproducibility. Table 1 shows the median height measurements for each replicate (median ± MAD), where MAD is the median absolute difference, as well as the mean and mean absolute difference (MAD) of the three experiments for 2 min, 3 h, and 5 h. The change from 2 min, 3 h, and 5 h is statistically significant (*p* < 0.01).

Control experiments using tau proteins (10 nM) in the bulk were conducted to evaluate the impact of the surface on aggregation kinetics (Figure S4). The number of aggregates in the bulk solution for 3 h (Figure S4a) was less compared to the images corresponding to the aggregation on mica for 3 h (Figure S4b), and this difference is illustrated by the data in Table S1, in which the number of aggregates depending on time is counted.

### 2.3. AFM Imaging of Aβ42 Aggregation

**Figure 2:**
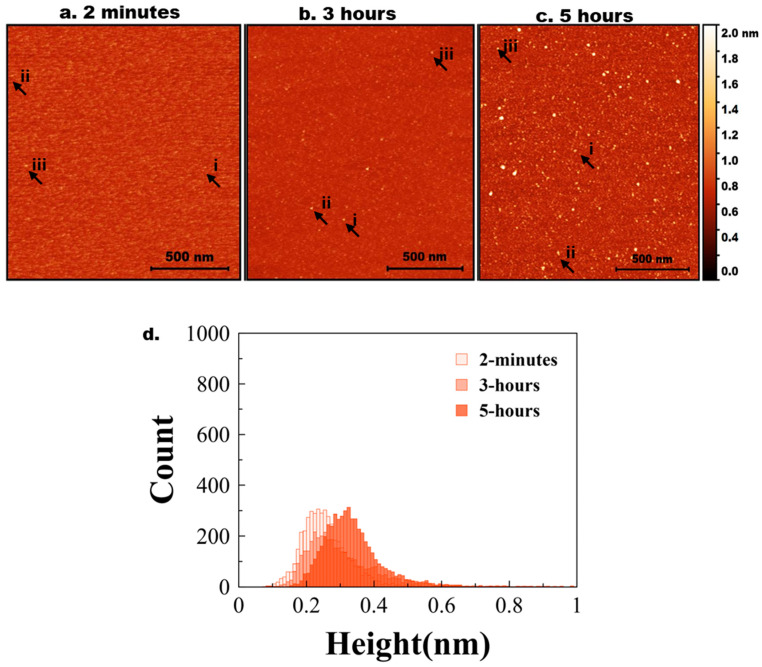
Experimental data for Aß42 aggregation. AFM topographical images of the on-surface aggregation of 10 nM Aß42 protein for (**a**) 2 min, (**b**) 3 h, and (**c**) 5 h. (**d**) Aβ42 height distribution for 2 min, 3 h, and 5 h with baseline shift.

Similar experiments were conducted for 10 nM Aβ42 aggregation, and the results are presented in Figure 2.

A few bright particles (indicated by arrows) corresponding to Aß42 aggregates, and this number grows over time (Figure 2b,c). Note, Aβ42 is 10 times smaller than the tau protein. The histograms corresponding to the particle size distribution for AFM images taken at 2 min, 3 h, and 5 h are shown in Figure 2d to illustrate the growth of particle sizes over time. Figure S8b shows a similar histogram with three different colors for clear representation. Three independent experiments were performed, and Figure S5 demonstrates the overlap plot of the height traces for 2 min and 5 h, providing a clearer visual representation of data reproducibility. Table 2 shows the median height measurements for each replicate (median ± MAD) of the three experiments for 2 min, 3 h, and 5 h. The change from 2 min to 5 h is statistically significant (*p* < 0.01).

### 2.4. AFM Imaging of Tau-Aβ42 Coaggregation

**Figure 3:**
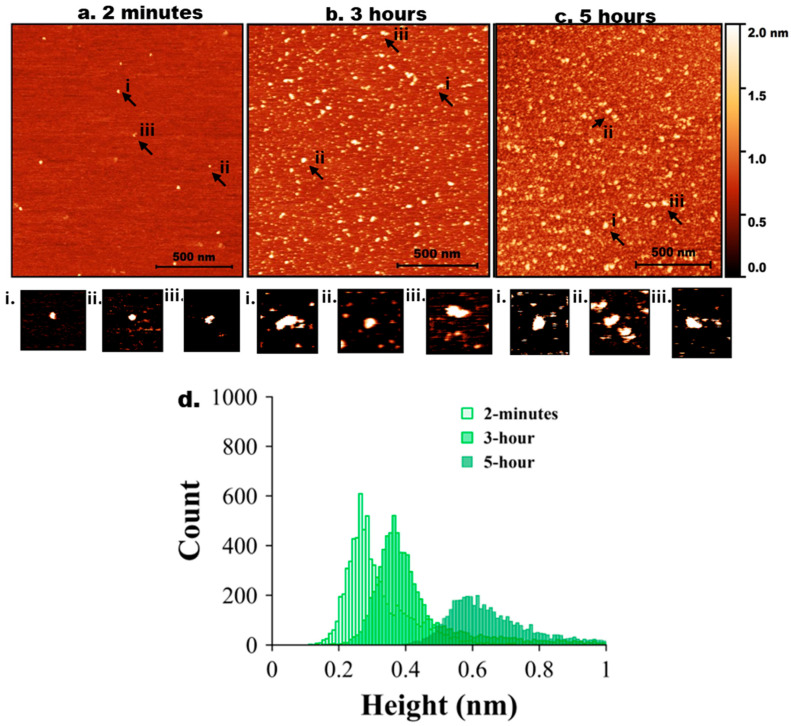
AFM imaging of tau-Aβ42 coaggregation. Representative AFM topographic images of tau-Aβ42 aggregates on mica surfaces at different incubation times. (**a**) 2 min, (**b**) 3 h, (**c**) 5 h. Arrows indicate representative aggregates. (**a**,**i**–**iii**) represents zoomed-in particles from 2 min. (**b**,**i**–**iii**) represents zoomed-in particles from 3 h. (**c**,**i**–**iii**) represents zoomed-in particles from 5 h. (**d**) Tau-Aβ42 height distribution for 2 min, 3 h, and 5 h with baseline shift.

Next, tau and Aβ42 proteins at 10 nM were mixed, and the experiment was repeated as described above for the individual proteins. AFM images are shown in Figure 3. Initially (2 min, Figure 2a), only a few particles of round shape were seen. A few zoomed-in images, indicated with arrows labelled i, ii, and iii, are shown below in Figure 3a. As incubation time increased to 3 h (Figure 3b), the number of tau-Aβ42 aggregates on the surface increased. Similarly, zoomed images of a few particles (i, ii, and iii) are shown below Figure 3b. These particles are visually larger than those in Figure 1b. The aggregation is more pronounced after 5 h of incubation (Figure 3c), as shown in a few zoomed-in images. Overall, these data demonstrate that the coaggregation of tau-Aβ42 is more pronounced than the aggregation of either tau or Aβ42 individually.

The height value analysis was conducted, and the results are presented in Figure 3d, which illustrates a significant shift in height values over time. Figure S8c shows a similar histogram with three different colors for clear representation. Three independent experiments were performed, and Figure S6 illustrates the overlap plot of the height traces for 2 min and 5 h, providing a clearer visual representation of data reproducibility. Table 3 shows the median height measurements for each replicate (median ± MAD) of the three experiments for 2 min, 3 h, and 5 h. The change from 2 min to 5 h is statistically significant (*p* < 0.01).

We added a negative control by mixing tau with two unrelated proteins of different sizes (Histone H1 and NF-κB). According to Figure S9, there is no effect on tau aggregation by these two proteins.

### 2.5. Comparative Analysis of AFM Image Data

**Figure 4:**
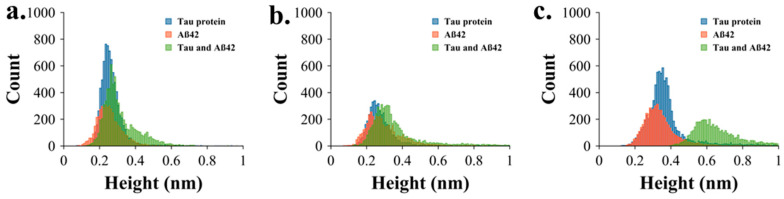
Comparative quantitative analysis of AFM images. (**a**) Overlapping height distribution of tau (blue), Aβ (orange), and tau & Aβ aggregates (green) at 2 min, (**b**) 3 h, and (**c**) 5 h.

Figure 4a–c provide a comparative aggregation profile by overlapping the height distributions of tau, Aβ42, and co-aggregated tau & Aβ42 at 2 min, 3 h, and 5 h. Blue, orange, and green traces represent tau, Aβ42, and tau & Aβ42, respectively. These overlays show that Aβ42 enhances tau aggregation, as the combined tau & Aβ42 samples consistently show higher heights, most notably at 5 h. The data assembled at this incubation time demonstrate that there is no overlap between the height distributions of aggregates assembled with the tau-Aβ42 mixture (green histogram) and those of the protein aggregated separately (orange and blue histograms). This finding suggests that the primary co-aggregation of these two proteins occurs more frequently than their independent aggregation.

To count the number of particles each hour, two separate frames were used for each hour, and the average is shown in Supplementary Table S2. For this particle counting, the volume cutoff for tau and Aβ42 was determined from the calibration curve [22] and used the approximate monomer size volume as a cutoff.

## 3. Discussion

**Figure 5:**
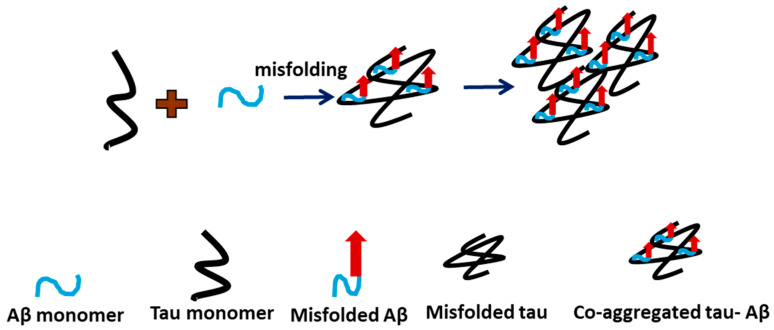
Graphical model of tau-Aβ42 interaction pathway. Co-aggregation of misfolded tau-Aβ occurs when mixed. Black indicates tau monomer, blue indicates Aβ monomer, and the blue with red arrow indicates misfolded Aβ.

Our studies revealed several novel features of tau aggregation and the role of Aβ42 in this process. First, we demonstrate that tau can aggregate at low nanomolar concentrations, suggesting that even small amounts of free tau in cells may be sufficient to initiate aggregation. In cells, tau is a structural protein present at concentrations of up to 2 μM as part of microtubules [23], but spontaneous dissociation can release free tau capable of forming aggregates [24,25]. Second, whereas most studies associate tau aggregation with phosphorylation, including hyperphosphorylation [26,27], our data demonstrate that unmodified, wild-type tau can aggregate, indicating that phosphorylation is not required for this process. Third, our findings suggest that Aβ42 catalyzes the aggregation of tau, as visually evident in the AFM images (Figure 3a–c). Importantly, quantitative analysis (Figure 4) further shows that these two proteins co-aggregate with no free Aβ42 aggregates found after 5 h (Figure 4c). These results imply that all Aβ42 is associated with tau aggregates. Hence, a 10-fold difference in size allows tau to accommodate several Aβ42 molecules, possibly in the oligomeric form. We posit that misfolded tau and Aβ42 interact to form a more aggregation-prone complex. Based on these findings, we propose the model in Figure 5 for tau aggregation in the presence of Aβ42, in which a direct interaction of Aβ42 with tau leads to the co-aggregation of tau and Aβ42, occurring more rapidly than free tau aggregation. Thus, the Aβ42-defined catalysis of tau aggregation is determined by the tau-Aβ42 assembly, leading to misfolding and aggregation that could contribute to the pathology process.

The significance of our findings to the pathological features of tau and Aβ42 in the AD development is discussed below.

The hallmarks of AD are senile plaques assembled primarily with Aβ42 and neurofibrillar tangles (NFT) formed by tau [11,28,29]. More recent experimental research suggests that Aβ promotes NFT development by facilitating either direct or indirect interactions with tau [18,30,31,32,33]. Our data directly show that Aβ42 catalyzes tau aggregation. Importantly, the tau aggregation is markedly enhanced by Aβ42, even at a low concentration of 10 nM. As previous studies show that it is within the range of the intracellular concentrations of soluble Aβ in neurons, this concentration is very significant [34].

The demonstration of tau protein aggregation in the absence of post-translational modifications, such as phosphorylation, is another crucial finding. Most in vitro tau aggregation studies employ chemical modifications, truncations, inducers, or mutations to promote fibrillization [16,35]. However, our findings demonstrate that full-length, unaltered tau can assemble on mica surfaces in a time frame comparable to that of Aβ42 under physiological conditions. While tau pathology in Alzheimer’s disease is commonly associated with hyperphosphorylation, our research suggests that tau has the inherent capacity to aggregate.

Extracellular tau oligomers have also been found to be pathologically active in Alzheimer’s disease, despite tau’s neurotoxicity traditionally being linked to its formation into intracellular neurofibrillary tangles. In animal models, short-term exposure to extracellular oligomeric tau reduces memory and hippocampal long-term potentiation (LTP), even when fibrillar deposits are absent [36]. The presence of extracellular Aβ is associated with plaque formation; however, these monomeric or oligomeric forms can also contribute to tau aggregation. In this instance, our demonstration of on-surface aggregation of Aβ42 and tau can be a mechanism that further catalyzes their co-aggregation in the presence of cell membranes and other organelles.

We have shown that phospholipid bilayers mimicking the cell membrane catalyze Aβ42 aggregation [37,38]; so, this mechanism can be applied to the tau-Aβ42 co-aggregation. The findings also suggest that tau may undergo conformational changes that facilitate self-assembly in specific microenvironments, such as intracellular compartments or near membrane interfaces. This supports the idea that phosphorylation may not be the exclusive focus in the early stages of tau aggregation, but that Aβ42 can play a critical role.

Using mica as a model surface enabled us to perform protein self-assembly experiments at physiologically relevant low nanomolar concentrations of Aβ42. To better replicate in vivo conditions, in future studies, we will use a supported lipid bilayer with different lipid components, including cholesterol, to better mimic physiological conditions.

## 4. Materials and Methods

### 4.1. Preparation of Protein Stock Solutions

Human recombinant wild-type protein tau-441 (2N4R), Catalog No. SPR-47, monomers were purchased from StressMarq Biosciences Inc., Victoria, BC, Canada. The lyophilized protein was reconstituted in 10 mM HEPES buffer (pH 7.4) containing 100 mM NaCl and stored at −80 °C. Working solutions were freshly prepared by serial dilution of the stock to achieve a final concentration of 10 nM in 10 mM HEPES buffer (pH 7.4) containing 100 mM NaCl.

Lyophilized Aβ42 peptide (Catalog No. RP10017; GenScript, Piscataway, NJ, USA) was prepared following a previously established protocol [37,39]. To eliminate any pre-existing aggregates, the peptide was first dissolved in 1,1,1,3,3,3-hexafluoroisopropanol (HFIP) in a glass vial to a final concentration of 50 μM. The HFIP was allowed to evaporate overnight at room temperature under a gentle nitrogen stream, leaving a thin peptide film. This film was then re-dissolved in DMSO to obtain a stock solution, which was stored at −20 °C until use. Before each experiment, an aliquot of the stock solution was dialyzed against 10 mM HEPES buffer (pH 7.4) containing 100 mM NaCl and subsequently diluted to a final protein concentration of 10 nM for use.

To prepare the reaction mixture, we mixed the stock solutions of tau, Aβ42, and their mixture in 10 mM HEPES buffer (pH 7.4) and adjusted the ionic strength to 100 mM NaCl.

### 4.2. Sample Preparation to Monitor On-Surface Aggregation

Freshly cleaved mica sheets were used as substrates for the on-surface aggregation assays. Small pieces of mica were incubated with tau, Aβ42, or tau-Aβ42 mixture solutions (10 nM final protein concentration) in Eppendorf tubes for up to 5 h. At defined time points (2 min, 3 h, and 5 h), the mica substrates were carefully removed, rinsed thoroughly with deionized water, dried under an argon stream, and finally pasted to a metal puck with sticky tape. The samples were then kept in a vacuum overnight to ensure complete drying before AFM imaging.

### 4.3. AFM Imaging and Analysis

AFM images were acquired in standard tapping mode under ambient conditions using a Multimode Nanoscope IV system (Bruker-Nano, Santa Barbara, CA, USA). TESPA probes with a nominal resonance frequency of 310–340 Hz and a spring constant of ~42 N/m were used for imaging. Before starting imaging, autotuning was performed in nanoscope software (version 6.14R1(R)). The scan rate was in the range of 1 to 1.5 Hz. Scan size was 1.5 × 1.5 µm^2^ with 1024 samples/line.

### 4.4. Image Analysis and Data Handling

Topographical images were plane-adjusted and flattened using a polynomial function in Gwyddion v2.66 (Gwyddion, Brno, Czech Republic). Direct quantification of particle size distribution becomes statistically complex due to the large degree of morphological heterogeneity. However, we can approximate aggregation through changes in baseline height. Intrinsically disordered proteins (IDPs), such as tau and AB42, are known to coat surfaces; here, we exploited this property to measure baseline height by comparing buffer-treated controls with Aβ42-treated samples. Supplementary Figure S7 displays control histograms (no protein, only buffer) and those with protein present for 2 min and 5 h. In Figure S7a, a clear baseline shift in height to the right is observed when protein is present, with gray representing control and orange representing Aβ42. Figure S4b shows a more pronounced baseline shift after 5 h, indicating increased aggregation. We acquired height traces in Gwyddion v2.66 (Gwyddion, Czech Republic), extracted the values in nm, and plotted the histograms in MagicPlot Pro v2.9.3 (St. Petersburg, Russia). The method is illustrated in the supplementary section (Figure S2). For each time point and sample type (tau, Aβ42, and their mixture), AFM images from 1.5 × 1.5 µm^2^ scan areas were analyzed.

For the statistical test, a two-sample *t*-test assuming unequal variances (Welch’s *t*-test) was conducted to compare the means of 2 min and 5 h across three different experiments involving tau, Aβ42, and tau & Aβ42. The analysis showed a significant difference between the two groups (*p* value (two-tailed)), indicating that the mean at 2 min was significantly lower than at 5 h in all the experiments mentioned above.

## 5. Conclusions

This work demonstrates that Aβ42 promotes and accelerates tau aggregation at physiologically low concentrations (10 nM). This can be a co-aggregation process in which the interaction between tau and Aβ42 drives aggregation. The finding that the process occurs at the surfaces of tau protein without modification suggests novel molecular mechanisms underlying the pathological effects of both physiologically essential proteins. Future research will explore tau-Aβ42 interactions on surfaces that more closely mimic the cellular environment, such as supported lipid bilayers.

## Figures and Tables

**Table 1:** Height trace measurement from tau aggregation at 2 min, 3 h, and 5 h.

Time	Exp1	Exp2	Exp3	Mean ± MAD
2 min	0.25 ± 0.05	0.32 ± 0.08	0.27 ± 0.00	0.28 ± 0.02
3 h	0.43 ± 0.28	0.29 ± 0.13	0.37 ± 0.12	0.36 ± 0.05
5 h	0.35 ± 0.20	0.40 ± 0.08	0.37 ± 0.39	0.37 ± 0.02

Exp 1, 2, and 3 display median ± MAD of the median.

**Table 2:** Height trace measurement from Aβ42 aggregation at 2 min, 3 h, and 5 h.

Time	Exp1	Exp2	Exp3	Mean ± MAD
2 min	0.25 ± 0.09	0.25 ± 0.02	0.30 ± 0.00	0.26 ± 0.02
3 h	0.30 ± 0.11	0.31 ± 0.12	0.28 ± 0.08	0.29 ± 0.01
5 h	0.32 ± 0.23	0.38 ± 0.11	0.30 ± 0.13	0.33 ± 0.03

Exp 1, 2, and 3 display median ± MAD of the median.

**Table 3:** Height trace measurement from tau-Aβ42 aggregation at 2 min, 3 h, and 5 h.

Time	Exp1	Exp2	Exp3	Mean ± MAD
2 min	0.29 ± 0.04	0.29 ± 0.02	0.29 ± 0.01	0.29 ± 0.00
3 h	0.41 ± 0.27	0.41 ± 0.26	0.48 ± 0.29	0.43 ± 0.03
5 h	0.64 ± 0.06	0.80 ± 0.10	0.80 ± 0.41	0.75 ± 0.07

Exp 1, 2, and 3 display median ± MAD of the median.

## Data Availability

The original contributions presented in this study are included in the article/Supplementary Materials. Further inquiries can be directed to the corresponding author.

## Supplementary Materials

The following supporting information can be downloaded at: https://www.mdpi.com/article/10.3390/ijms262412128/s1.

## Abbreviations

The following abbreviations are used in this manuscript:

AFM	Atomic force microscopy
Aβ	Amyloid beta
AD	Alzheimer’s Disease
nM	Nanomolar
NFT	Neurofibrillary Tangle
MAD	Mean/Median Absolute Difference

## References

[1] Kumar A., Sidhu J., Lui F., Tsao J.W. (2024). Alzheimer Disease. StatPearls.

[2] Subedi S., Sasidharan S., Nag N., Saudagar P., Tripathi T. (2022). Amyloid Cross-Seeding: Mechanism, Implication, and Inhibition. Molecules.

[3] Rajmohan R., Reddy P.H. (2017). Amyloid Beta and Phosphorylated Tau Accumulations Cause Abnormalities at Synapses of Alzheimer’s Disease Neurons. J. Alzheimers Dis..

[4] Parihar M.S., Brewer G.J. (2010). Amyloid Beta as a Modulator of Synaptic Plasticity. J. Alzheimers Dis..

[5] Ciechanover A., Kwon Y.T. (2015). Degradation of Misfolded Proteins in Neurodegenerative Diseases: Therapeutic Targets and Strategies. Exp. Mol. Med..

[6] Kent S.A., Spires-Jones T.L., Durrant C.S. (2020). The Physiological Roles of Tau and Aβ: Implications for Alzheimer’s Disease Pathology and Therapeutics. Acta Neuropathol..

[7] Ashraf G.M., Greig N.H., Khan T.A., Hassan I., Tabrez S., Shakil S., Sheikh I.A., Zaidi S.K., Wali M.A., Jabir N.R. (2014). Protein Misfolding and Aggregation in Alzheimer’s Disease and Type 2 Diabetes Mellitus. CNS Neurol. Disord. Drug Targets.

[8] Guerrero-Muñoz M.J., Gerson J., Castillo-Carranza D.L. (2015). Tau Oligomers: The Toxic Player at Synapses in Alzheimer’s Disease. Front. Cell Neurosci..

[9] Hurtado D.E., Molina-Porcel L., Iba M., Aboagye A.K., Paul S.M., Trojanowski J.Q., Lee V.M.Y. (2010). Aβ Accelerates the Spatiotemporal Progression of Tau Pathology and Augments Tau Amyloidosis in an Alzheimer Mouse Model. Am. J. Pathol..

[10] Tofigh N., Agahi S., Riazi G., Ghalamkar Moazzam M., Shahpasand K. (2025). A Novel Phosphorylated Tau Conformer Implicated in the Tauopathy Pathogenesis of Human Neurons. Biomolecules.

[11] Zhang H., Wei W., Zhao M., Ma L., Jiang X., Pei H., Cao Y., Li H. (2021). Interaction between Aβ and Tau in the Pathogenesis of Alzheimer’s Disease. Int. J. Biol. Sci..

[12] Ittner L.M., Götz J. (2010). Amyloid-β and Tau—A Toxic Pas de Deux in Alzheimer’s Disease. Nat. Rev. Neurosci..

[13] Vasconcelos B., Stancu I.C., Buist A., Bird M., Wang P., Vanoosthuyse A., Van Kolen K., Verheyen A., Kienlen-Campard P., Octave J.N. (2016). Heterotypic Seeding of Tau Fibrillization by Pre-Aggregated Abeta Provides Potent Seeds for Prion-like Seeding and Propagation of Tau-Pathology in Vivo. Acta Neuropathol..

[14] Karran E., De Strooper B. (2022). The Amyloid Hypothesis in Alzheimer Disease: New Insights from New Therapeutics. Nat. Rev. Drug Discov..

[15] Clinton L.K., Blurton-Jones M., Myczek K., Trojanowski J.Q., LaFerla F.M. (2010). Synergistic Interactions between Abeta, Tau, and Alpha-Synuclein: Acceleration of Neuropathology and Cognitive Decline. J. Neurosci..

[16] Zhang W., Falcon B., Murzin A.G., Fan J., Crowther R.A., Goedert M., Scheres S.H.W. (2019). Heparin-Induced Tau Filaments Are Polymorphic and Differ from Those in Alzheimer’s and Pick’s Diseases. eLife.

[17] Nam Y., Shin S.J., Kumar V., Won J., Kim S., Moon M. (2025). Dual Modulation of Amyloid Beta and Tau Aggregation and Dissociation in Alzheimer’s Disease: A Comprehensive Review of the Characteristics and Therapeutic Strategies. Transl. Neurodegener..

[18] Miller Y., Ma B., Nussinov R. (2011). Synergistic Interactions between Repeats in Tau Protein and Aβ Amyloids May Be Responsible for Accelerated Aggregation via Polymorphic States. Biochemistry.

[19] Lazarevic V., Fieńko S., Andres-Alonso M., Anni D., Ivanova D., Montenegro-Venegas C., Gundelfinger E.D., Cousin M.A., Fejtova A. (2017). Physiological Concentrations of Amyloid Beta Regulate Recycling of Synaptic Vesicles via Alpha7 Acetylcholine Receptor and CDK5/Calcineurin Signaling. Front. Mol. Neurosci..

[20] Pan Y., Banerjee S., Zagorski K., Shlyakhtenko L.S., Kolomeisky A.B., Lyubchenko Y.L. (2019). Molecular Model for the Surface-Catalyzed Protein Self-Assembly. J. Phys. Chem. B.

[21] Banerjee S., Hashemi M., Lv Z., Maity S., Rochet J.C., Lyubchenko Y.L. (2017). A Novel Pathway for Amyloids Self-Assembly in Aggregates at Nanomolar Concentration Mediated by the Interaction with Surfaces. Sci. Rep..

[22] Ratcliff G.C., Erie D.A. (2001). A Novel Single-Molecule Study to Determine Protein−Protein Association Constants. J. Am. Chem. Soc..

[23] Avila J. (2010). Intracellular and Extracellular Tau. Front. Neurosci..

[24] Zheng H., Sun H., Cai Q., Tai H.C. (2024). The Enigma of Tau Protein Aggregation: Mechanistic Insights and Future Challenges. Int. J. Mol. Sci..

[25] Iqbal K., Liu F., Gong C.-X., Grundke-Iqbal I. (2010). Tau in Alzheimer Disease and Related Tauopathies. Curr. Alzheimer Res..

[26] Šimić G., Babić Leko M., Wray S., Harrington C., Delalle I., Jovanov-Milošević N., Bažadona D., Buée L., de Silva R., Di Giovanni G. (2016). Tau Protein Hyperphosphorylation and Aggregation in Alzheimer’s Disease and Other Tauopathies, and Possible Neuroprotective Strategies. Biomolecules.

[27] Liu M., Sui D., Dexheimer T., Hovde S., Deng X., Wang K.W., Lin H.L., Chien H.T., Kweon H.K., Kuo N.S. (2020). Hyperphosphorylation Renders Tau Prone to Aggregate and to Cause Cell Death. Mol. Neurobiol..

[28] Deture M.A., Dickson D.W. (2019). The Neuropathological Diagnosis of Alzheimer’s Disease. Mol. Neurodegener..

[29] Nelson P.T., Braak H., Markesbery W.R. (2009). Neuropathology and Cognitive Impairment in Alzheimer Disease: A Complex but Coherent Relationship. J. Neuropathol. Exp. Neurol..

[30] Eckert A., Hauptmann S., Scherping I., Rhein V., Müller-Spahn F., Götz J., Müller W.E. (2008). Soluble Beta-Amyloid Leads to Mitochondrial Defects in Amyloid Precursor Protein and Tau Transgenic Mice. Neurodegener. Dis..

[31] Hauptmann S., Keil U., Scherping I., Bonert A., Eckert A., Müller W.E. (2006). Mitochondrial Dysfunction in Sporadic and Genetic Alzheimer’s Disease. Exp. Gerontol..

[32] David D.C., Hauptmann S., Scherping I., Schuessel K., Keil U., Rizzu P., Ravid R., Dröse S., Brandt U., Müller W.E. (2005). Proteomic and Functional Analyses Reveal a Mitochondrial Dysfunction in P301L Tau Transgenic Mice. J. Biol. Chem..

[33] Younas N., Saleem T., Younas A., Zerr I. (2023). Nuclear Face of Tau: An inside Player in Neurodegeneration. Acta Neuropathol. Commun..

[34] Ripoli C., Cocco S., Li Puma D.D., Piacentini R., Mastrodonato A., Scala F., Puzzo D., D’Ascenzo M., Grassi C. (2014). Intracellular Accumulation of Amyloid-β (Aβ) Protein Plays a Major Role in Aβ-Induced Alterations of Glutamatergic Synaptic Transmission and Plasticity. J. Neurosci..

[35] Rasmussen H.Ø., Nielsen J., de Poli A., Otzen D.E., Pedersen J.S. (2023). Tau Fibrillation Induced by Heparin or a Lysophospholipid Show Different Initial Oligomer Formation. J. Mol. Biol..

[36] Taddei R.N., Perbet R., De Gerando A.M., Wiedmer A.E., Sanchez-Mico M., Stewart T.C., Gaona A., Melloni A., Amaral A.C., Duff K. (2023). Tau Oligomer–Containing Synapse Elimination by Microglia and Astrocytes in Alzheimer Disease. JAMA Neurol..

[37] Banerjee S., Hashemi M., Zagorski K., Lyubchenko Y.L. (2021). Cholesterol in Membranes Facilitates Aggregation of Amyloid β Protein at Physiologically Relevant Concentrations. ACS Chem. Neurosci..

[38] Hashemi M., Banerjee S., Lyubchenko Y.L. (2022). Free Cholesterol Accelerates Aβ Self-Assembly on Membranes at Physiological Concentration. Int. J. Mol. Sci..

[39] Banerjee S., Hashemi M., Zagorski K., Lyubchenko Y.L. (2020). Interaction of Aβ42 with Membranes Triggers the Self-Assembly into Oligomers. Int. J. Mol. Sci..

