# Supplementary material for: Catalytic Effect of Amyloid-β on Native Tau Aggregation at Physiologically Relevant Concentrations
Source: Int J Mol Sci. 2025 Dec 17;26(24):12128. doi: 10.3390/ijms262412128 (PMC12733284; doi:10.3390/ijms262412128)
Supplement: Supplementary file 1 [file ijms-26-12128-s001.zip › ijms-3939342-supplementary.pdf]

Catalytic Effect of Amyloid-β on Native Tau Aggregation at Physiologically Relevant Concentrations

Rakhi Chowdhury [1], Apu Chandra Das[2], Ruan van Deventer[1], Luda S Shlyakhtenko[1] and Yuri L Lyubchenko [1*]

Supplementary Figures

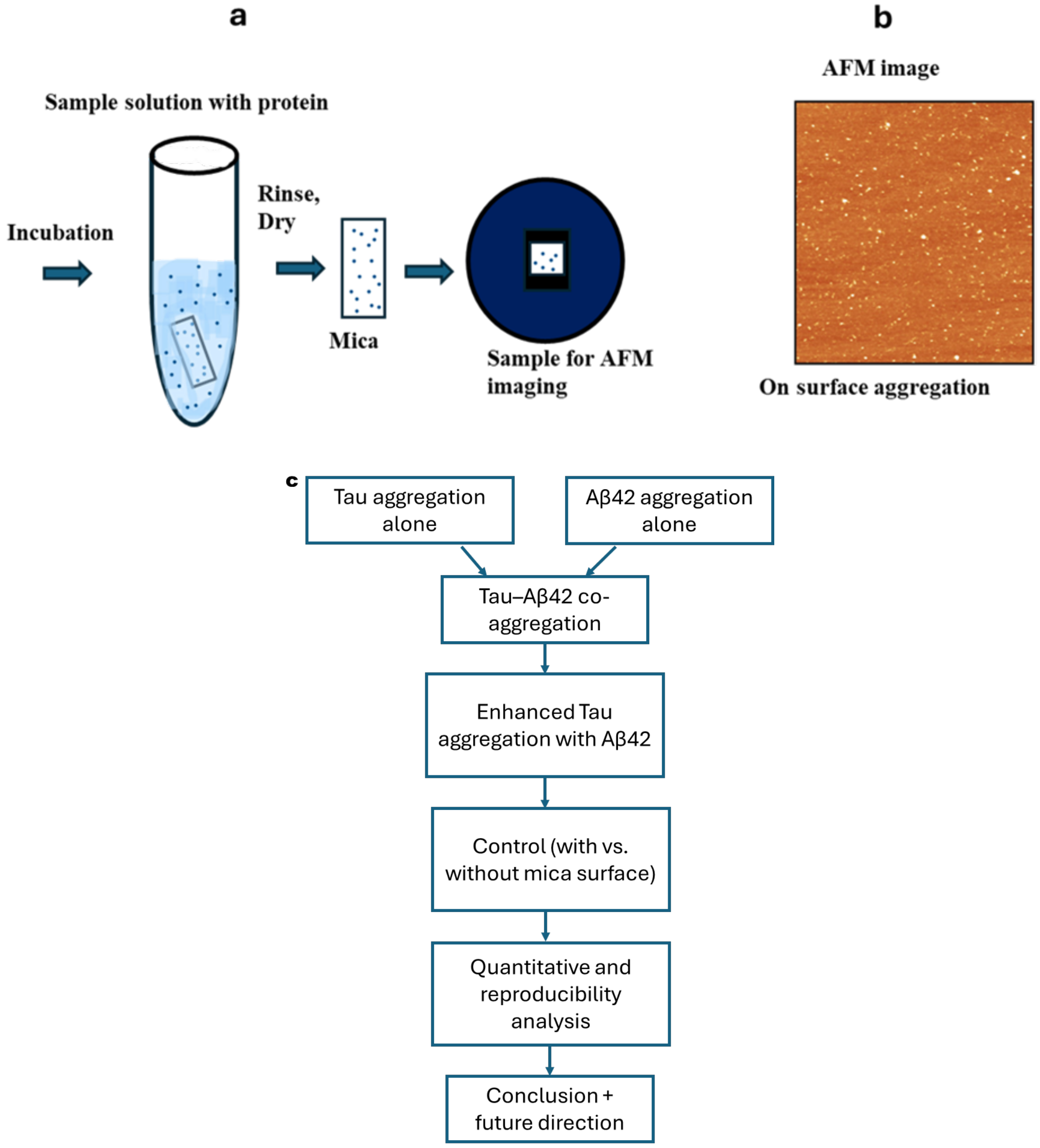

Figure S1. (a) Schematic presentation of the experimental setup of the surface mediated aggregation of a protein (b) AFM image of aggregates assembled on surface. (c) Flow and outcome of the experiments.

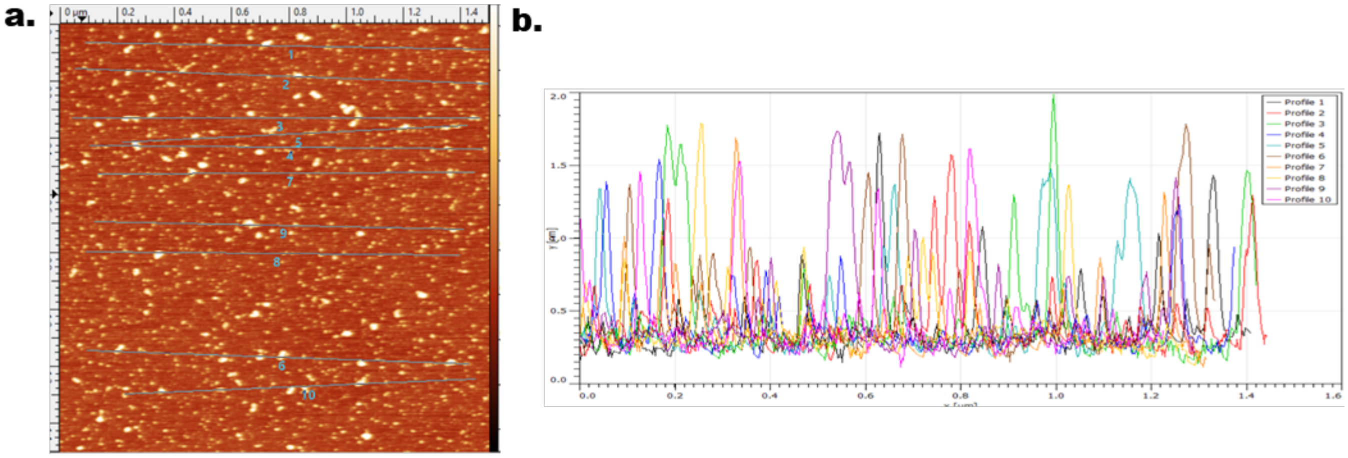

Figure S2. Data acquisition method. (a) AFM image with a set of lines along which the height values were measured. (b) Corresponding peak from the lines acquired from the lines. The different colors in (b) represent individual line traces from 1-10 (a).

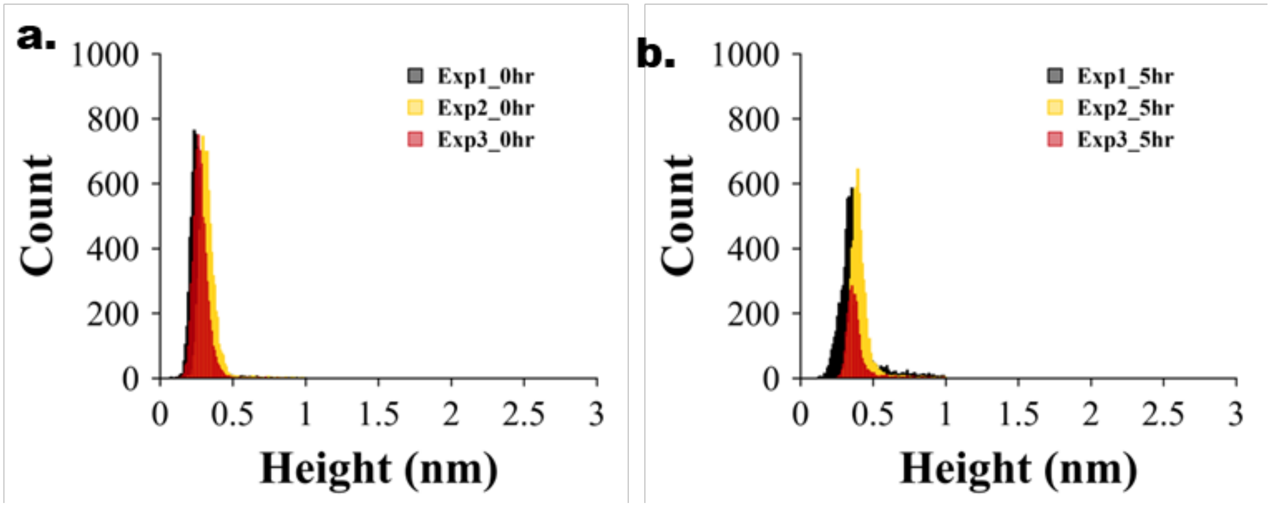

Figure S3. Histograms for tau height values for three independent experiments for different times. (a) 2 minutes and (b) 5 hours.

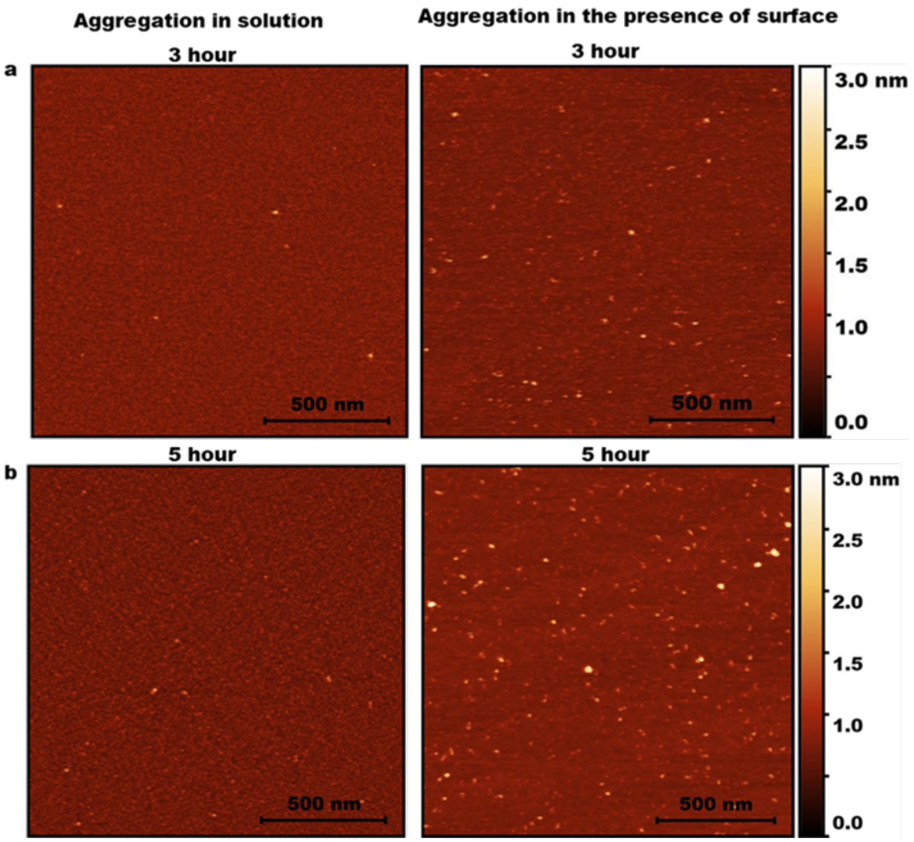

Figure S4. AFM images for tau aggregated in the bulk solution (left rows) and on mica (right rows) corresponding to (a) 3 hours and (b) 5 hours.

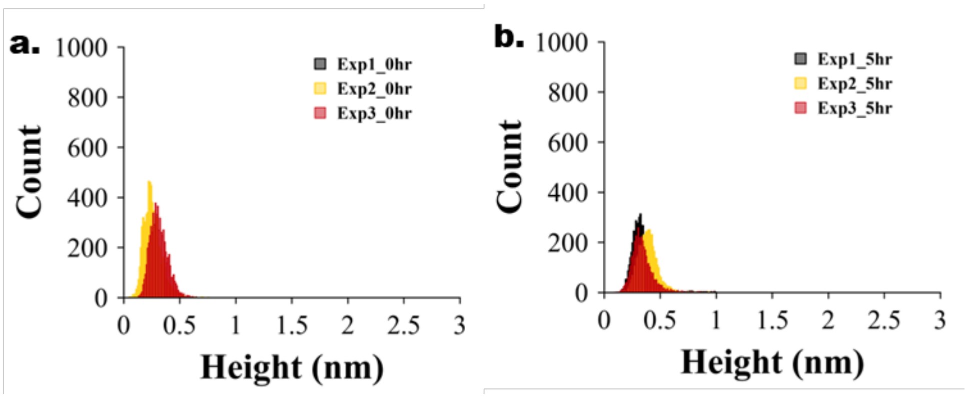

Figure S5. Histograms for Aβ42 height values for three independent experiments at different times. (a) 2 minutes and (b) 5 hours.

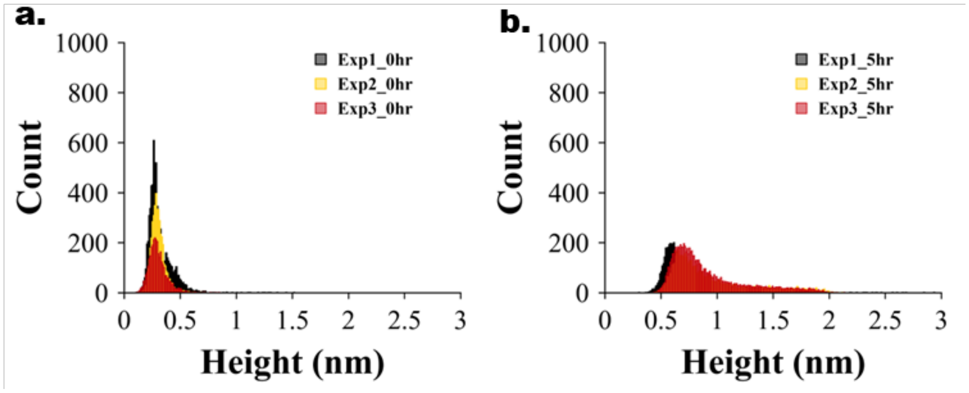

Figure S6. Histograms of height values for the aggregation experiments for the mixture of tau and Aβ42. Different colors correspond to the data for each experiment for (a) 2 minutes and (b) 5 hours.

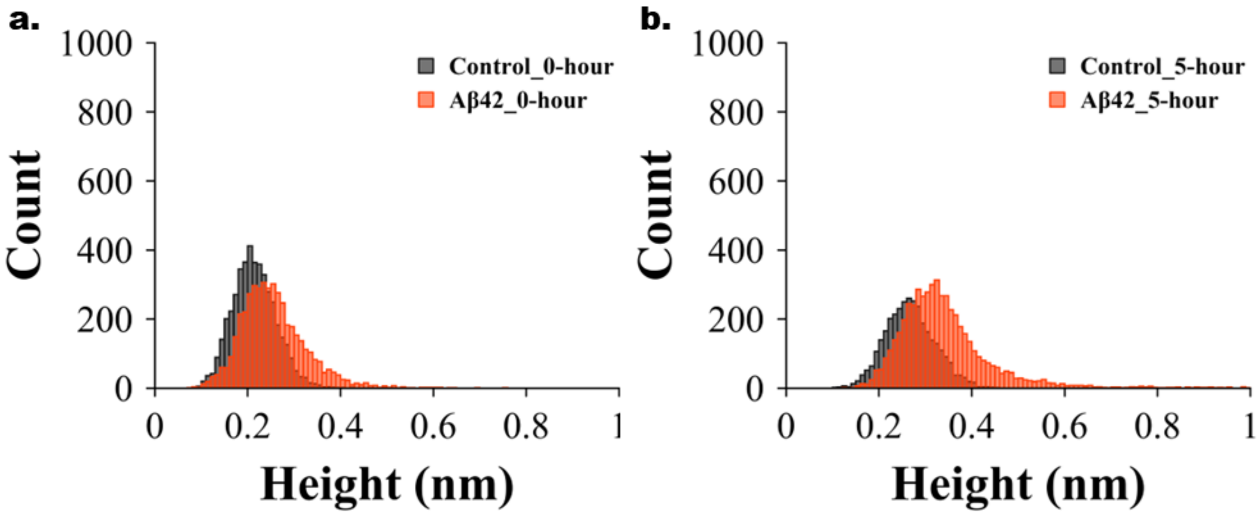

Figure S7. The height shifts in the presence of protein. (a) Control vs Aβ42 height distribution at 2 minutes. (b) Control vs Aβ42 height distribution at 5 hours.

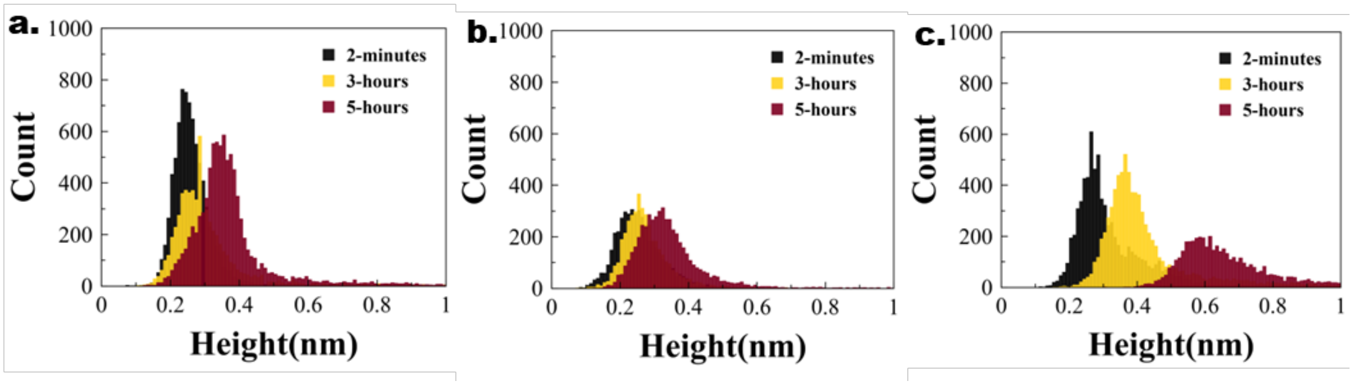

Figure S8. Height distribution for 2 minutes, 3 hours, and 5 hours with baseline shift. (a) Tau, (b) Aβ42, and (c) tau & Aβ42.

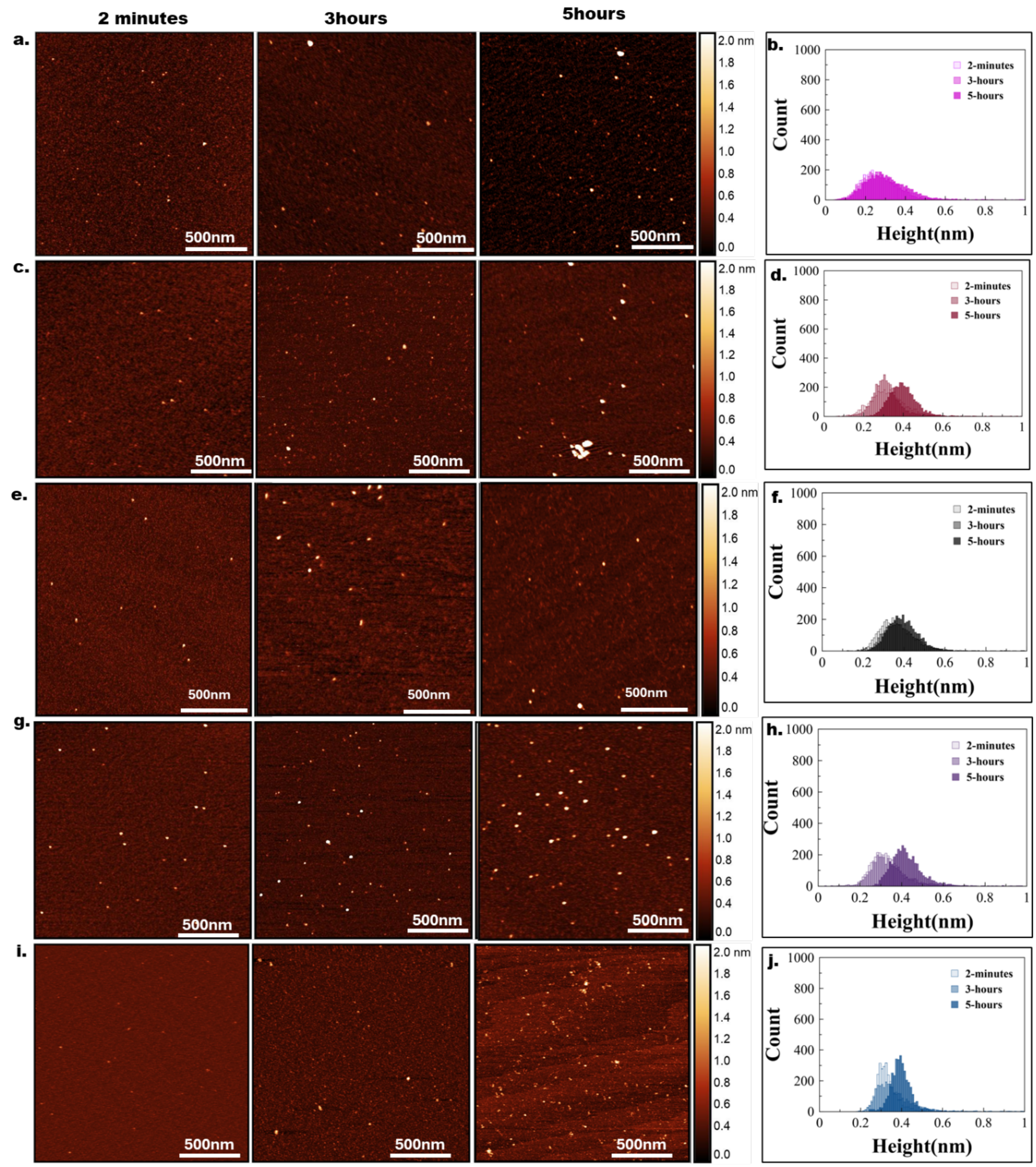

Figure S9. AFM imaging of H1, Tau-H1, NF-κB, and Tau-NF-κB coaggregation on mica surfaces at different incubation times of 2 minutes, 3 hours, and 5 hours. (a) H1 aggregation, (b) H1 height distribution for 2 minutes, 3 hours, and 5 hours with baseline shift, (c) Tau-H1 aggregation, (d) Tau-H1 height distribution for 2 minutes, 3 hours, and 5 hours with baseline shift, (e) NF-κB aggregation, (f) NF-κB height distribution for 2 minutes, 3 hours, and 5 hours with baseline shift, (g) Tau-NF-κB aggregation, (h) Tau-NF-κB height distribution for 2 minutes, 3 hours, and 5 hours with baseline shift, (i)Tau aggregation, (j) Tau height distribution for 2 minutes, 3 hours, and 5 hours with baseline shift.

Table S1. Average number of particles for tau protein in the bulk solution and on the surface aggregation at 3 and 5 hours.

Time of incubation	Number of particles Bulk solution	Number of particles On surface
3hour	49	129
	49	119
**mean**	**49**	**124**
5hour	210	366
	146	236
**mean**	**178**	**301**

Table S2. The time dependence of the number of particles for experiments with tau, Aβ42, and tau-Aβ42.

	**2minutes**	**3hour**	**5hour**
**Tau**	21	119	366
	22	129	236
**Aβ42**	179	438	773
	375	846	1040
**Tau & Aβ42**	145	935	1599
	105	915	1589

The number of particles was calculated from two different frames from a single experiment, using a cutoff value based on the expected volume for tau and Aβ42.

Table S2 shows the number of particles increased over time for all proteins. However, there is a significant increase in the number of particles for the tau-Aβ42 mixture, which is more pronounced at longer incubation times, 5 hours.
